# Donor-Derived Cell-Free DNA in Allograft Transplantation: Exaggerated Hope or Cautious Reality?

**DOI:** 10.3390/biomedicines13102325

**Published:** 2025-09-23

**Authors:** Marina Fernández-González, Santiago Llorente, Carmen Botella, José Antonio Galián, Rosana González-López, María José Alegría-Marcos, Alicia Hita, Rosa Moya-Quiles, Helios Martínez-Banaclocha, Manuel Muro-Pérez, Javier Muro, Alfredo Minguela, Isabel Legaz, Manuel Muro

**Affiliations:** 1 Immunology Service, University Clinical Hospital “Virgen de la Arrixaca”, Biomedical Research Institute of Murcia (IMIB), 30120 Murcia, Spain; marina.fernandez3@carm.es (M.F.-G.); mcarmen.botella@carm.es (C.B.); josea.galian3@carm.es (J.A.G.); rosana.gonzalez@carm.es (R.G.-L.); mariaj.alegria@carm.es (M.J.A.-M.); alicia.hita@carm.es (A.H.); rosa.moya2@carm.es (R.M.-Q.); helios.martinez2@carm.es (H.M.-B.); muro_manuel@live.com (M.M.-P.); javimuro12@gmail.com (J.M.); alfredo.minguela@carm.es (A.M.); 2 Nephrology Service, University Clinical Hospital “Virgen de la Arrixaca”, Biomedical Research Institute of Murcia (IMIB), 30120 Murcia, Spain; santiagoj.llorente@carm.es (S.L.); 3 Department of Legal and Forensic Medicine, Biomedical Research Institute of Murcia (IMIB), Faculty of Medicine, Regional Campus of International Excellence “Campus Mare Nostrum”, University of Murcia, 30100 Murcia, Spain

**Keywords:** acute rejection, allograft rejection, biomarker, chronic kidney disease, donor-derived cell-free DNA, tolerance.

## Abstract

Nowadays, there have truly been spectacular advances in surgical techniques, the preservation of organs for transplants, the optimal and efficient selection of both donors and recipients, a more efficient diagnosis and prediction of possible complications of transplants, and important progress in the advances of pharmacological immunosuppression protocols and procedures. In this sense, survival rates after transplantation of various organs have been progressively increasing, especially in the case of lung transplants, whose average survival rate is usually lower than that of other types of solid organ transplants. Thus, detecting acute and subclinical rejection and chronic allograft rejection of any implant is important. This is important in all transplants, such as heart and lung transplants. In this last type of transplant, particularly, and due to the chronic dysfunction of the lung allograft, it is key to detect rejection early and on time, since it can reach close to half of the transplant patient population. Therefore, practical diagnostic tools are needed to visualize the level of allograft damage using genomic methods such as those that measure donor-derived cell-free DNA, where its amount increases in the plasma component of the transplant after tissue injury or due to allograft infection. This biomarker has become a key element with light and hope, but with some shadows of caution due to its use as a panacea. Our research team has experience in solid organ transplantation in quantifying this parameter in the progression of the lesion of the implanted allograft, and our experience and comparison with the published literature will be presented in the following review, discussing validated and non-validated results.

## 1. Background

Classically, in the various types of transplants, the biopsy of the grafted organ is usually the reference method for adequate monitoring. In this sense, the biopsy consists of taking a small sample of the transplanted tissue and sending it to a pathological anatomy service for microscopic examination, thus evaluating and guaranteeing the viability of the allograft. The biopsy is performed to detect any evidence or signs of possible rejection or other problems that may arise in the transplanted organ. However, like any detection method that depends on the subjective evaluation of the human eye, in addition to being invasive and potentially causing discomfort to the patient. The majority of these methods are not absolute, risk-free, nor ideal. In this sense, it has been described that approximately 1% of biopsies may present complications, with a risk of macroscopic hematuria greater than 3.5% [1], although these tend to be generally mild and localized (pain and bleeding). However, there are other more serious complications, such as the risk of organ perforation, which could even lead to severe bleeding, sepsis, and death.

Consequently, we must strive to achieve safety profiles that minimize the risk of post-transplant complications with more specific procedures [2].

Fortunately, better detection of subclinical rejection has avoided the need for routine biopsies, and new non-invasive clinical biomarkers have been developed that allow obtaining more information about the status of the allograft and, if necessary, detecting rejection to proceed more promptly, increase or modify the immunosuppression administered, and implement anti-rejection therapies [3].

These new non-invasive biomarkers that allow the detection of subclinical rejection include urinary and serum markers such as biochemical formulas or ratios (urinary albumin-creatinine ratio), the detection of donor-specific antibodies (DSA), the presence of specific cell populations that vary during rejection, or more powerful imaging techniques (magnetic resonance imaging and ultrasound) to detect changes in the graft that could indicate rejection [4,5,6,7,8]. Likewise, at the genomic level, we have an important range of promising biomarkers: gene expression (serum and in mTOR biopsies), differential expression of microRNA [9], analysis of mitochondrial DNA or analysis of circulating free DNA derived from the donor (dd-cfDNA) that can help in the diagnosis of subclinical rejection and allow early intervention [10,11,12,13].

## 2. New Biomarkers, Non-Invasive in Transplant as dd-cfDNA

An interesting new biomarker, dd-cfDNA, has been the subject of numerous studies in a relatively short period. This non-invasive biomarker monitors solid organ transplants like kidney, heart, or lung transplants.

Although dd-cfDNA increases its concentration in plasma when tissue damage occurs in the graft, demonstrated by multiple studies over the last 10 years, and could be an important biomarker; however, alterations in the percentage or quantity of dd-cfDNA are not a truly specific biomarker of a rejection event, because other pathologies or events of tissue damage also increase these numbers, such as acute tubular necrosis or BK virus infection [14,15,16].

However, during these years, the actual ability of dd-cfDNA as a biomarker has been highlighted in many studies [17,18,19,20,21,22,23] that give it a very positive role in evaluating the optimal state of the graft and as a predictor of its good or bad evolution. This determination can be performed as a commercial type with several reference providers (more widespread in the USA), where they determine the patient samples in their laboratories or by performing it in the patients’ own hospitals using optimized in-house commercial kits, using NGS techniques, even with alternative methods [23].

However, several interesting questions arise that should be answered in this present review.

The first issue to clarify is the definition of dd-cfDNA. To answer it, we must know that, after an organ transplant, part of the donor’s cfDNA circulates in the recipient’s bloodstream and that an increase in dd-cfDNA levels can indicate organ injury or rejection.

Another question would be: what is its actual clinical use? To answer this second question, we must know that dd-cfDNA helps monitor acute rejection, is used with traditional markers such as creatinine or biopsy, and is common in monitoring kidney, heart, and lung transplants.

**Figure 1:**
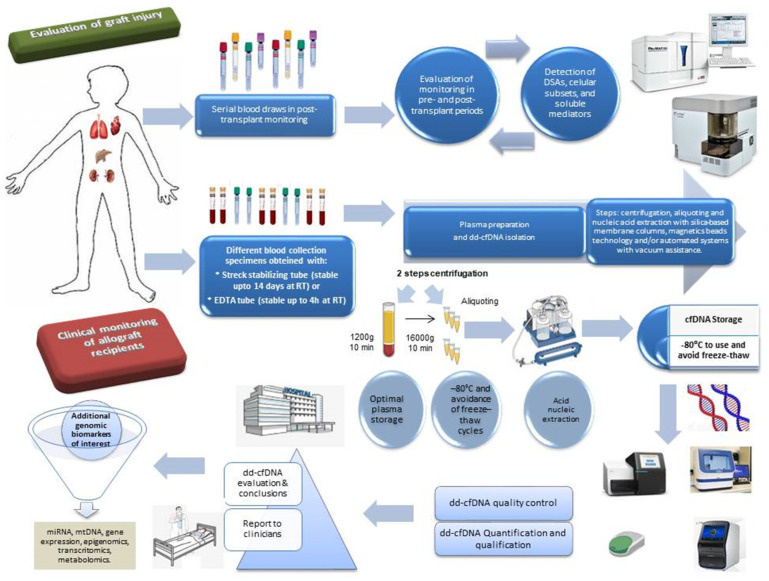
Example of post-transplant monitoring of different biomarkers in allograft recipients, especially dd-cfDNA testing. Several important steps are shown. Samples of blood, urine, or any organ-specific biological fluid are collected in regular laboratory tubes, depending on the biomarker (DSA test and soluble mediator dry serum tube, EDTA or heparin tube for cell markers and cultures) and stabilizing tubes (Streck or EDTA), where the stability time is different. In the case of cfDNA extraction, plasma preparation with two steps centrifugation, acid nucleic extraction (with silica-based membrane column, magnetic beads technology and/or automated systems with vacuum assistance) and cfDNA isolation (with storage), quality control of the extracted cfDNA, analysis via NGS, digital PCR, or qPCR, and subsequent reporting of the dd-cfDNA result are carried out. The different result is communicated from the laboratory to the clinical professional who evaluates it and could indicate and modify the immunosuppressive treatment. They can also be performed, if the results are inconclusive or show a need for expansion with other genomic biomarkers (miRNA, mtDNA, gene expression, epigenomics and transcriptomics, etc.), cellular (regulatory and functional populations) or soluble mediators (monitoring of DSA, cytokines, chemokines or soluble proteins, metabolomics, etc.) to evaluate and predict the future prognosis of the allograft recipient. Abbreviations: cfDNA, cell free DNA; dd-cfDNA, donor-derived cell free DNA; miRNA, microRNA; mtDNA, mytocondrial DNA; RT, room temperature; DSA, donor specific antibodies; PCR, polymerase chain reaction; qPCR, quantitative PCR.

The third question is: what are some examples of different dd-cfDNA tests? Nowadays, there are various methods and techniques for the determination and quantification of dd-cfDNA, which measures graft damage and offers a real possibility for the non-invasive recognition of allogeneic damage in transplant recipients, in addition to classic determinations such as AlloSure^®^ (CareDx) (Brisbane, CA, USA), Prospera^®^ (Natera) (Austin, TX, USA), or TRAC^®^ (Viracor Eurofins) (Lenexa, KS, USA) [24,25]. In the Figure 1, we will see how this dd-cfDNA biomarker can be quantified.

## 3. How Can dd-cfDNA Be Determined?

Several methods for the determination and quantification of dd-cfDNA will be discussed below. There are two primary and most used methods for its quantification: random and targeted techniques, each with advantages for detecting dd-cfDNA in transplant recipients. Random techniques use adapter ligation followed by next-generation sequencing (NGS). Targeted techniques typically involve the use of single-nucleotide polymorphisms (SNPs), droplet digital PCR (ddPCR), or quantitative PCR (qPCR).

Developed by Beck et al., in 2013 [26], a single PCR sample is divided into 20,000 individual droplets in ddPCR. This partitioning of target molecules into droplets creates a highly accurate quantification method without standard curves. This method demonstrates greater precision than qPCR technique, especially when low levels of DNA are available [3,27]. DdPCR results are also known to have a shorter turnaround time of 1 day in contrast to NGS results, which can take 2 to 3 days [28].

The most used method is NGS technique for the quantification of dd-cfDNA, in which, for each transplant patient, Streck tubes are extracted, kept at room temperature, and centrifuged within 7 days after sample collection. The plasma obtained from centrifugation (the first should be 1600× *g* for 20 min, and the second at 16,000× *g* for 10 min) should be stored at −80 °C until further analysis. The cfDNA must be extracted from the plasma with different commercial kits (for example, the QiAmp circulating nucleic acid kit (Qiagen, Dusseldorf, Germany)) and always follow the manufacturer’s instructions, since it is a delicate material. Subsequently, NGS evaluates the relative amount of dd-cfDNA with more than 200 SNP polymorphisms, preparing the libraries with the AlloSeq cfDNA Assay. (CareDx, Brisbane, CA, USA) Following the manufacturer’s protocol and performing sequencing on a MiSeq instrument using the 300-cycle MiSeq Micro v2 or 150-cycle MiSeq v3 kits (Illumina, San Diego, CA, USA), depending on the number of samples to be processed. Data analysis is performed with AlloSeq cfDNA v2.2.1 software (CareDx), and dd-cfDNA is quantified as the percentage of all the cfDNA available in the sample [21].

## 4. Studies of dd-cfDNA in Kidney Transplantation

Historically, in kidney transplants, serum creatinine, glomerular filtration rate, and urinary markers were the only ones used to measure kidney function and evaluate the graft status due to their low cost, simple procedure, straightforward interpretation, and relative reliability. However, its sensitivity and specificity for detecting allograft damage are minimal. Therefore, the true and only current definitive method for the diagnosis of rejection in kidney transplant patients is the graft biopsy [12] and it continues to be the reference method for monitoring transplanted kidneys, from early detection to routine screening (which is becoming less frequent), as well as to evaluate the correct or incorrect progression of the allograft [13,21].

However, being a clinically invasive method, it will cause different problems of safety, discomfort, tolerability, and discrepancy in acceptability for transplantation. In this sense, almost 1% of the biopsies performed will cause certain complications [1].

Furthermore, with the different current immunosuppressants that are administered, the correct detection of subclinical rejection is unfortunately still rare, which will always lead us to look for other non-invasive methods to measure the renal allograft function. Furthermore, it is important to recognize that the sensitivity and specificity to measure kidney graft damage with biopsy are also, on different occasions, limited.

Consequently, there are also other non-invasive procedures, especially in kidney and heart transplants, for the monitoring of transplant recipients with rejection, such as the possibility of monitoring the detection of HLA and non-HLA antibodies, models of gene expression and differential expression of specific miRNAs, different tests for monitoring cellular immunity, differential evaluation of the levels of specific urinary chemokines (CXCL9 or CXCL10), or also the development of different signatures or proteomic models and peptides to be able to evaluate the appearance and development of rejection in monitoring urine and peripheral blood samples [6,9,12,13,29], although none of these different biomarkers developed in the literature has reached the peak of a complete validation. These biomarkers have also not reached a complete extensive and consistent scientific demonstration. These have also not demonstrated accurate and helpful support and economic support within the healthcare field where resources are quite limited. In this sense, some of these biomarker evaluation procedures are quite expensive, not immediate in definition of results and/or to some extent cumbersome for routine use in clinical practice. Therefore, searching for and evaluating new biomarkers in kidney transplantation is essential.

In this sense, the introduction of dd-cfDNA has emerged as a more precise non-invasive biomarker to quantify tissue damage and injury, and more preventive for the diagnosis of eventual graft rejection compared to other classic biomarkers and, if its use is extended, costs are lowered and the process is further streamlined, it may become that promising and dreamed biomarker with great potential for the correct and rapid early detection of kidney graft damage [21]. Thus, the objective of early identification of increased levels of dd-cfDNA concentration could even make it unnecessary to perform the biopsy and could also allow the modification, by appropriate evaluation of the results, of the immunosuppressive therapy.

In this sense, there are already very numerous and extensive studies, individual single-center and even multicenter, that evaluate this biomarker in the monitoring and evolution of the renal implant, such as Allosure with CareDx, Prospera with Natera and Trac with Viracor-Eurofins [21,24,25,30], and which also exhibit cut-off levels that allow us to discriminately distinguish and define whether we are in actual renal rejection (dd-cfDNA > 1%) or not, as well as the evaluation of how we can be sure that there is good sensitivity, reasonable specificity, and that allow us to define the negative predictive values (NPV) and know that we can be calm regarding the correct evolution of the graft and the positive predictive values (PPV) of the quantification of dd-cfDNA that should lead us to take therapeutic measures.

In this regard, a multicenter study of dd-cfDNA by Bloom et al. [22] prospectively analyzed biopsy samples and found that dd-cfDNA levels discriminated between biopsy samples showing some rejection (TCMR or AMR) and samples without histological rejection. The PPVs and NPVs for active rejection at a cutoff of 1.0% dd-cfDNA were 61% and 84%, respectively, and the AUC to discriminate between AMR and non-AMR samples was 0.87. The PPVs and NPVs for AMR with a cutoff value of 1.0% dd-cfDNA were 44% and 96%, respectively. On the other hand, the median dd-cfDNA resulted in 2.9% for AMR, 1.2% for TCMR types ≥ IA and IB), 0.2% for type IA TCMR, and 0.3% in controls.

In a relatively recent study, our clinical and healthcare research group presented the results of our kidney transplant patients in which the concentration levels of dd-cfDNA were evaluated during 2 years of follow-up [21] and where we observed that 60% of patients presented positive or elevated values, coinciding with the diagnosis by biopsy and the detection of DSA antibodies, as in other studies already mentioned [18,31]. However, we report that this biomarker resembles cellular rejection better than antibody-mediated rejection, unlike the group of Cuchiari et al. [15,23].

Other studies, however, such as the one carried out by the group of Aubert et al. [32] show that high levels of dd-cfDNA correlate with the occurrence and severity of all types of renal graft rejection, including subclinical, even in recipients with stable allograft function, where a high NPV of the biomarker made it possible to avoid having to perform biopsies.

In our experience, however, this assumption is a risk since our results demonstrate that a low NPV, due to the possible impact of other pathologies or post-transplant biological complications (obesity, recurrent autoimmune diseases and infections) on dd-cfDNA levels, should be taken into account when interpreting the results [21], and dd-cfDNA cases can be considered false positives.

On the other hand, the group of Aubert et al. [32] also demonstrated an association with subclinical rejection in recipients with stable renal allograft function and suggests that this biomarker could constitute a valid diagnostic tool with real potential. Additionally, the group of Punukollu et al. [33] suggests in a good review that the function of dd-cfDNA and the gene expression profile (GEP) allow the adequate identification of acute rejection, as well as other emerging biomarkers such as Torque-techno virus (TTV), which has shown potential as an indirect indicator of immunosuppression levels and the risk of rejection. Along the same lines, other authors [32] have shown that the addition of dd-cfDNA, combined with conventional kidney transplant markers, increases the discriminatory capacity of the diagnosis of rejection.

Another interesting study by Cuadrado-Payan et al. [23] includes graft biopsies per protocol and indication. The median dd-cfDNA in each diagnostic group was 0.40% in borderline rejection, 0.60% in TCMR, 1.48% in AMR, and 0.33% without rejection. Cases with DSA+ and rejection had higher dd-cfDNA levels compared to DSA+ cases without rejection, and dd-cfDNA levels showed an association with microvascular inflammation and C4d positivity. The AUC of dd-cfDNA to discriminate any rejection from its absence was 0.74, and excluding borderline rejection, 0.80, surpassing other markers of kidney function. Within TCMRs, there were no differences in dd-cfDNA levels between IA and ≥IB rejections. There were also no differences between acute and chronic-active ABMR. Cases with DSA+ and rejection data on biopsy showed higher levels of dd-cfDNA than cases without DSA+ but without rejection, with a median of 2.13% versus 0.69%. Furthermore, cases with DSA+ and ABMR in biopsy also presented a higher median dd-cfDNA (2.34%) vs. ABMR cases without DSA (0.97%). In the univariate analysis, the variables associated with rejection were the biopsy indication, the presence of DSA, previous transplant, and dd-cfDNA levels. In the multivariate analysis, the indication biopsy, the DSA at the time of the biopsy, and the dd-cfDNA levels maintained their association.

Finally, in the association analysis of dd-cfDNA with the individual scores of the Banff classification, a correlation was observed with microvascular inflammation and the presence of C4d+, but not with tubulointerstitial inflammation.

In summary, there are differences in dd-cfDNA levels between patients with and without rejection, being considerably higher in those with rejection, especially in cases of ABMR, suggesting that dd-cfDNA could be more sensitive in the detection of ABMR compared to TCMR or borderline rejection [20,21,22,23,31], underlining the potential of dd-cfDNA to identify rejection non-invasively. This contrasts with other studies that have reported higher dd-cfDNA levels in severe TCMR rejections (≥ IB) [32,34]. These contradictory data could be attributed to the heterogeneity in the presentation and severity of rejection, to the fact that dd-cfDNA does not entirely reflect tissue damage, or to the fact that the pathophysiological mechanisms do not generate detectable amounts of cfDNA in circulation, in addition to the small sample size of patients with TCMR in the different studies.

There are more studies on the behavior of dd-cfDNA in ABMR [35,36,37], showing higher levels in patients with ABMR than in those with DSA+, but without damage in the biopsy. Integrating these two types of information (dd-cfDNA and presence or absence of DSA) would allow us to discern whether or not the immunological phenomenon would be causing damage to the graft. These data reflect that dd-cfDNA could have limitations in detecting borderline rejection [22,38,39], although in these cases, it could help identify patients with a worse prognosis in terms of renal function and rejection progression [34].

Comparatively, the diagnostic performance of dd-cfDNA appears superior to that of other markers of renal function, reaffirming the value of dd-cfDNA as a more sensitive and specific marker for acute rejection in kidney transplant recipients.

Moreover, regarding the analysis of dd-cfDNA in relation to the individual scores of the Banff classification, it supports its usefulness to evaluate ABMR, since there seems to be an association with microvascular inflammation and CD4 positivity, but not with tubulointerstitial inflammation, as previously described [23,40,41,42].

However, most studies have important limitations. First, the lack of longitudinal monitoring of dd-cfDNA levels after biopsy and rejection treatment prevents the evaluation of its usefulness as a dynamic, prognostic, or response to treatment biomarker. Previous studies suggest that changes in post-treatment levels could indicate the therapeutic response and graft recovery [20,38,40]. Furthermore, the heterogeneity of rejection contemplated in many studies may influence the interpretation of the results.

Finally, with the current method to quantify dd-cfDNA, it is challenging to differentiate the proportion corresponding to other previous non-functioning grafts, an element that must be expanded with more cases evaluated in the literature. The knowledge generated should allow us to discern the area of use of dd-cfDNA in different clinical situations [34,40,43].

Finally, in a recent article of Nie et al. [44] and combining dd-cfDNA fraction and absolute quantification improves diagnostic accuracy for kidney transplant rejection, especially antibody mediated rejection where the double-positive and double-negative approaches showed high predictive value, offering potential clinical value for monitoring kidney transplant recipients.

Therefore, in conclusion, plasma levels of dd-cfDNA could be considered a promising alternative biomarker for monitoring graft rejection in the early stages of kidney transplantation, up to several months before its clinical presentation and enhances its value in the detection of acute renal rejection, although further more studies are required to estimate the actual significant difference in plasma levels of dd-cfDNA in terms of differentiating cellular or humoral rejection.

## 5. Studies of dd-cfDNA in Liver Transplantation

In liver transplantation, as in the kidney transplant that we just mentioned, liver biopsy continues to be the reference method to evaluate various allograft complications, despite the possible associated risks.

As in kidney transplantation, the emergence and validation of new biomarkers for the early detection and prediction of liver graft damage, without the potential risks of biopsy, should play a fundamental role and have a significant real clinical impact.

In this sense, the technology to detect dd-cfDNA is a promising tool to sensitively detect dysfunction of the transplanted liver graft [24].

Various published articles also show that the levels of the dd-cfDNA biomarker were correlated with the ratio of aspartate aminotransferase to alanine aminotransferase [45,46], demonstrating good diagnostic performance in the evaluation of acute rejection. Likewise, another recent prospective cohort study suggests that the dd-cfDNA biomarker by SNP and NGS outperformed conventional liver function tests in appropriately predicting acute rejection [47].

Furthermore, a very recent study in a Japanese population by Kanamori et al. [48] characterized the dynamics of the different levels of dd-cfDNA after liver transplantation. It evaluated its use for correctly monitoring the integrity of the allograft and the appropriate diagnosis of acute rejection. Additionally, dd-cfDNA levels align with liver allograft fibrosis and C4d scores to evaluate the potential for its use as a general indicator of appropriate allograft integrity. The diagnostic accuracy of SNP-targeted dd-cfDNA in this study demonstrated an AUC of 0.975 for the development of acute rejection. The quantification of dd-cfDNA showed a sensitivity and specificity of 100% and 90.9%, respectively, for the diagnosis of acute rejection, providing an NPV of 100%, which suggests avoiding invasive biopsy when dd-cfDNA is below the cut-off value [48].

On the other hand, in theory, the destruction of donor liver tissue releases two copies of dd-cfDNA into the blood, derived from damage to cellular sources, such as endothelial cells or other liver stromal cells [24].

An additional aspect regarding cfDNA is that its short half-life (minutes to 1.5 h) continuously reflects allograft damage in a real-time mode, while biochemical estimation of liver function tests has long half-lives, which limits the optimal and appropriate real ability to monitor the different continuous changes that occur in the liver graft.

However, a fundamental limitation in the case of liver transplantation is the elevated levels of dd-cfDNA associated with active viral and bacterial infections, as well as other vascular and biliary complications [24,25].

In conclusion, this may be the preferred evaluation for recipients with abnormal liver function tests, implying benefit in detecting rejection and in prospective trials to minimize administered immunosuppression.

The absence of elevated dd-cfDNA levels and routine liver function tests could eliminate the need for biopsies in the future. Finally, samples with suspected chronic rejection (fibrosis with inflammation and C4d) may have persistently elevated levels of dd-cfDNA [47].

This year’s very recent and novel study by McNamara et al. [48] provides the monitoring of 130 blood samples from 44 patients at different times after liver transplantation for dd-cfDNA methylation.

Sequential methylation of dd-cfDNA fragments was mapped into an atlas of cell type-specific DNA methylation patterns, derived from 476 purified cell methylomes. DNA methylation patterns and multi-omics data integration for liver cell types showed distinctive enrichment in open chromatin and functionally important regulatory regions. These authors observed that post-transplant multitissue cellular damage recovered in patients without allograft injury during the first postoperative week. However, the sustained elevation of cfDNA in hepatocytes and biliary epithelium during the first month indicated early-onset allograft injury. Furthermore, the composition of the cfDNA differentiated the causes of allograft injury, which may indicate, in the future, if confirmed by subsequent studies from other groups and/or larger studies from the same group, the real potential for monitoring allograft status and non-invasive intervention.

## 6. Studies of dd-cfDNA in Heart Transplantation

Firstly, heart transplantation is usually the final treatment for end-stage heart failure, allowing patients undergoing this procedure to live longer and with a better quality of life after the intervention [49]. However, there are still problems in this type of transplant, despite the better surgical procedures, the improvement of pharmacological immunosuppression protocols, and a more appropriate and adequate management of the post-transplant evolution of the patient undergoing a cardiac implant.

As in other types of transplants we have seen previously, one of the main immune problems in heart transplantation is rejection, which occurs when the recipient’s immune system perceives and recognizes the grafted heart as foreign and triggers an inflammatory response to attack it.

We must try to understand what mechanisms promote rejection of the heart graft or, conversely, its acceptance and tolerance to update and implement new developments, procedures and protocols that increase safety and the prediction of a more favorable evolution of the grafted heart and, if rejection occurs, to be able to manage and treat it more quickly and effectively. We must also assume that this is a more complex transplant than a kidney or liver transplant, since, in the event of graft failure after a heart transplant, the patient’s death is more likely, which can occur quickly if it is not retransplanted or connected to a mechanical circulatory assistance device. Therefore, the immunological rejection of this type of transplant must be stricter regarding its diagnosis, monitoring, and effective immunosuppressive treatment.

In particular, the types of rejection that can occur in the cardiac graft are ACR, AMR, or cardiac allograft vasculopathy (CAV). Identifying appropriate biomarkers of any of these processes detrimental to the cardiac graft, such as genomic markers, such as dd-cfDNA quantification, will facilitate patient management. Next, we will develop knowledge about these tools and procedures in heart transplantation.

Regarding the diagnosis of rejection, endomyocardial biopsy (EMB) appears to be useful, but new approaches and less invasive methods are needed to evaluate the immune status of the allograft. Among these non-invasive methods, there are developments such as miRNA expression analysis, Luminex determination of DSAs, dd-cfDNA levels, the release of specific soluble immune molecules, or the elaboration of gene expression profiles that are already operational and in validation studies to evaluate the tolerance or rejection status of the cardiac allograft [50,51].

In this sense, it is logical to think that, with the destruction of the cardiomyocytes of the allograft, associated with a possible rejection event, small fragments of the donor’s DNA are released that end up in the blood circulation of the cardiac recipient.

Analyzing the various publications on heart transplantation, a first study [52] analyzed 565 plasma samples to quantify the effectiveness of dd-cfDNA in detecting graft rejection. The results revealed that the sensitivity and specificity of the biomarker dd-cfDNA levels were comparable to those of EMB. Another later report shows an increase in circulating levels of dd-cfDNA immediately after transplantation, but this decreases to 0.13% at 28 days [53]. These disparate data indicate that dd-cfDNA levels stabilize at basal levels one week after implantation [54,55].

However, it is sometimes tricky to reliably associate heart transplant clinical data with specific biomarkers, although NPV can be obtained. In this sense, dd-cfDNA levels correlate with the degree of acute rejection, with a high NPV, increasing when a rejection event occurs and decreasing when the anti-rejection treatment is successful [53,56].

However, other factors can confuse when considering dd-cfDNA levels as determinants to define rejection, and they should be considered with caution. These particular factors, which can lead to certain elevated levels of dd-cfDNA without the presence of cardiac rejection, include myocardial ischemia, myocardial trauma, pregnancy, and multiorgan transplantation [57].

In addition to the data mentioned above, other groups report that, in more than 20% of cases, serum dd-cfDNA levels may be elevated, but cardiac recipients do not present symptoms or clinical signs of rejection [58].

Even today, the method to quantify plasma levels of dd-cfDNA is complex because it does not allow us to determine whether cardiac rejection is cellular or mediated by antibodies. Therefore, while the results may be promising in this particular type of transplant, in the current situation, more studies and additional validated results will be necessary to confirm and reliably implement this diagnostic procedure as a definitive method to monitor tolerance or rejection in recipients undergoing heart transplant [59].

A very recent interesting study using droplet digital PCR to analyze dd-cfDNA concentrations (measured in copies/mL) and fractional abundance (%dd-cfDNA) as a function of SNP polymorphism homozygosity was performed in 77 patients [60]. Both of these biomarkers, mean dd-cfDNA (cp/mL) and %dd-cfDNA, showed similar decreasing trends after heart transplantation and higher levels of dd-cfDNA (cp/mL) and %dd-cfDNA during acute rejection episodes compared to non-rejection samples. Furthermore, AMR appears to be associated with higher %dd-cfDNA levels than patients without rejection and ACR. In the logistic regression, %dd-cfDNA appeared as an early predictor of the risk of AMR between 10 and 19 days post-transplant, setting the %dd-cfDNA threshold at 0.125% to detect AMR. In summary, the authors estimate that early post-transplant %dd-cfDNA measurements can define patients with a low risk of having AMR in the first year after cardiac implantation [60].

Finally, another interesting and very recent review that explores the pathophysiological basis and clinical utility of dd-cfDNA in monitoring of heart transplant recipients with particular focus on evaluation and management of discordant findings has been reported [61]. As stated in this review and other significantly updated review for heart transplantation [62], more studies and validation of dd-cfDNA levels are needed for its implementation as a single reference for graft status.

## 7. Studies of dd-cfDNA in Lung Transplant

Finally, lung transplant differs from other transplants, such as kidney transplants, because it is a vital therapeutic option and its long-term results are determined by chronic lung allograft dysfunction (CLAD), which is the leading cause of post-transplant mortality [63]. This is an irreversible deterioration in lung function, defined as a decrease in forced expiratory volume in the first second (FEV1) ≥ 20% compared to baseline [64].

Classically, there are two main phenotypes of CLAD: bronchiolitis obliterans syndrome (BOS) and restrictive allograft syndrome (RAS).

The first type of CLAD, BOS, shows a decrease in FEV1 ≥ 20% from baseline with an obstructive pulmonary function test (PFT), in the absence of persistent radiological opacities or decreased total lung capacity. The second type of CLAD, RAS, is characterized by a decrease in FEV1 ≥ 20% compared to baseline, accompanied by a restrictive pattern in pulmonary function tests (a decrease in total lung capacity greater than or equal to 10% compared to baseline) and persistent opacities on chest x-ray or computed axial tomography (CAT) due to interstitial or pleural fibrosis [63].

The early stages of allograft dysfunction may manifest as changes in small airway function or lung structure, as evidenced by other diagnostic modalities, despite maintaining stable FEV1 levels (known as preclinical CLAD). There is a 2001 International Society for Heart and Lung Transplantation consensus document on diagnosing BOS [64].

Standard allograft monitoring usually includes periodic clinical evaluations, pulmonary function tests, imaging studies such as chest x-rays or CAT, and periodic bronchoscopies with transbronchial biopsies and bronchoalveolar lavage (BAL), essential to diagnose rejection, infection, and other post-transplant complications [63].

However, like other types of transplants, invasive biopsy will carry risks such as bleeding and pneumothorax, and is prone to sampling errors. Pulmonary function tests can also be affected by factors such as obesity, diaphragmatic dysfunction, patient exertion, etc. [63], making accurate estimation of lung allograft function difficult.

Therefore, sensitive and non-invasive biomarkers such as dd-cfDNA are needed to evaluate graft lung function. The values to evaluate allograft damage can range, as in other types of transplants discussed above, between 0.5% and 1% [21].

Very little is known about how dd-cfDNA levels may affect different degrees of CLAD and its progression. However, some studies report increases in dd-cfDNA in CLAD and its relationship with the deterioration of FEV1 [65], corroborated by other studies [66,67,68,69]. However, these trials failed to distinguish between CLAD, BOS, and RAS types.

It is also important to consider that the percentage of dd-cfDNA can be altered by several parameters, depending on when in the post-transplant period we make the determination, the calculation methods, or the development of an infectious process [68,70]. A recently published study exploring the role of plasma dd-cfDNA levels as a biomarker of CLAD over time (at three consecutive time points, without infection, without acute rejection, during stable CLAD, preclinical and established CLAD) [63] showed elevated levels of dd-cfDNA in 47% of stable CLAD, 66% of preclinical CLAD and 71% of CLAD samples, indicating a continuous lesion of the lung allograft, although with a high intra- and inter-patient variability, suggesting that more studies are needed to consider this interesting biomarker as fully established and validated in the diagnosis of lung damage, as is rightly suggested in a very recent reported review [71].

## 8. Future Directions and dd-cfDNA in Therapeutic Approaches in Transplantation

Currently, after everything stated in this review, we conclude that the use of dd-cfDNA methods is gradually gaining more and more importance as a diagnostic tool and as a biomarker of the function of the transplanted organ, mainly due to its sensitivity and specificity, which allow early and reliable detection of possible damage to the allograft. These quantifying concentrations, percentages, or methylation states of dd-cfDNA are easy to use and interpret, do not require excessive sample manipulation, and can be performed with a single sample [63,72,73].

In general, in the case of organ damage or dysfunction, dd-cfDNA levels increase rapidly, allowing for faster and more effective treatment of subclinical rejection than with traditional and classical biochemical measures. This reduces the number of unnecessary biopsies and allows physicians to modify immunosuppressive therapy promptly, preventing the progression of tissue damage, thereby reducing costs and improving patient outcomes. However, there are still factors of doubt and how different parameters of the donor and recipient can influence the quantification of dd-cfDNA and alter the results, as well as the lack of consensus on what clinical data should be taken into account, and agree on whether the absolute value or the percentage of dd-cfDNA should be standardized with respect to improving patient management [24,30].

Other important limitations, especially in the case of the United States, include using commercial kits that require sending the recipient’s blood samples to outside laboratories, resulting in results that often differ little from clinical findings. cfDNA is somewhat unstable, and during transport, if specific tubes are not used, the result will be altered, which generally does not occur in European transplant centers [21,74].

One of the most critical aspects of this technique, primarily if it is performed using NGS technique, with respect to healthcare systems, is its high cost, which makes its true clinical implementation in post-transplant routines considerably and relatively difficult [75].

Therefore, its future implementation is subject to solving problems and very important limitations related to economics (these procedures are not cheap and their cost is expensive), logistics (in the case of external shipment of samples without local procedure), variability (comparisons could be difficult between different results, and also with respect to different patients heterogeneity and the need of improving technical standardization is also required), improving validation (different results in different transplant situation and different patients in different types of transplantation), accurately demonstrating validity (in the sense of that these results should have clinical implication to improve transplant outcome), and achieving a stable and definitive international consensus. Furthermore, the possibility of longitudinal follow-up for personalized immunosuppression should also be addressed and this true limitation is also very important to monitor transplant outcome [17,76,77].

Finally, more research is needed to ensure that dd-cfDNA quantification becomes a safe, validated, and reliable tool to predict the early onset of graft damage, allowing modification of immunosuppression and administration of appropriate treatment to optimize outcomes in transplant recipients.

In conclusion, dd-cfDNA quantification is a promising biomarker for organ transplant rejection, but more studies are required for its full implementation in the post-transplant follow-up of transplant recipients.

Future research will be required to fully understand the role of dd-cfDNA in allograft rejection fully. However, it is necessary to mention that in dd-cfDNA studies, there is still a lack of proper standardization in data normalization methods and a gold standard and reliable endogenous control. These different studies tell us that transplant patients with rejection present differences in dd-cfDNA compared to tolerant transplants without rejection, indicating that these biomarkers could diagnose rejection in peripheral blood, urine samples, or cells isolated from organ transplant recipients. It is recommended that future trials, evaluations, and studies be carried out to corroborate and apply these interesting results in clinical practice, thus avoiding annoying invasive allograft biopsies.

## Abbreviations

DSA	Donor specific antibodies
cfDNA	Cell free DNA
dd-cfDNA	Donor-derived cfDNA
ddPCR	Droplet digital PCR
HLA	Human Leucocyte Antigen
SNP	Single Nucleotide Polymorphism
NGS	Next Generation sequencing
TCMR	T cell mediated rejection
AMR	Antibody mediated rejection
PPV	Positive predictive value
NPV	Negative predictive value
IFN	Interferon
IL	Interleukin
CAV	Cardiac allograft vasculopathy
EMB	Endomyocardial biopsy
CLAD	chronic lung allograft dysfunction
FEV	Forced expiratory volume
RAS	Restrictive allograft syndrome
BOS	Bronchiolitis obliterans syndrome
ACR	Acute cellular rejection
PFT	Pulmonary function test
CAT	Computed axial tomography
mtDNA	Mityocondrial DNA
miRNA	MicroRNA
RT	Room Temperature
BAL	Bronchoalveolar lavage
TTV	Torque-Technovirus
